# Wearable-Enhanced mHealth Intervention to Promote Physical Activity in Manual Wheelchair Users: Single-Group Pre-Post Feasibility Study

**DOI:** 10.2196/70063

**Published:** 2025-06-05

**Authors:** Zijian Huang, Dan McCoy, Rosemarie Cooper, Theresa M Crytzer, Yueyang Chi, Dan Ding

**Affiliations:** 1 Department of Rehabilitation Science and Technology, School of Health and Rehabilitation Sciences, University of Pittsburgh, 6425 Penn Ave, Suite 401, Pittsburgh, PA, 15206, United States, 1 4126241964; 2 Department of Veteran Affairs Pittsburgh Healthcare System, Human Engineering Research Laboratories, Pittsburgh, PA, United States; 3 Department of Physical Therapy, John G Rangos Sr School of Health Sciences, Duquesne University, Pittsburgh, PA, United States; 4 Department of Psychiatry, School of Medicine, University of Pittsburgh, Pittsburgh, PA, United States

**Keywords:** spinal cord injury, community-based research, exercise, workout, telemedicine, telerehabilitation, mHealth

## Abstract

**Background:**

With the rapid advancement of technology, using wearable devices and mobile health (mHealth) apps to monitor and promote physical activity (PA) has become increasingly popular among individuals with various chronic conditions. However, such work remains limited among individuals with spinal cord injury (SCI), especially those who use a manual wheelchair for mobility.

**Objectives:**

The study aims to describe the development of the WheelFit mHealth app for monitoring and promoting PA in manual wheelchair users (MWUs) with SCI and evaluate its feasibility and usability in free-living conditions.

**Methods:**

The WheelFit app, based on the Fogg Behavioral Model with inputs from stakeholders, including MWUs, physical therapists, and personal trainers, was developed to promote PA in MWUs. It works with two commercial wearable devices, that is, an Android smartwatch and a wheel sensor, which stream users’ upper extremity and wheelchair movement to the app to calculate PA variables using custom algorithms. Users can set personal goals, review daily progress and PA history, and access an adaptive workout library within the app. A 4-week single-group pre-post study, consisting of a 1-week baseline and 3-week intervention phase, was conducted to evaluate WheelFit’s feasibility and usability. Feasibility was evaluated using the session attendance rate, device and app usage, and implementation of action plans. Usability was assessed using the system usability scale. The preliminary effectiveness was assessed by comparing preintervention and postintervention PA variables and scores from the SCI exercise self-efficacy scale.

**Results:**

A total of 16 participants completed the study protocol with 100% session attendance and maintained 14.2 hours of daily device and app connection. Participants demonstrated varying levels of adherence to their action plans. The excellent usability of WheelFit was indicated by an average system usability scale score of 81.8 (SD 19.2) points. Statistically significant increases between pre-post daily exercise times (preintervention: mean 26.4, SD 16.9 minutes; postintervention: mean 33.3, SD 24.9 minutes; *P*=.049) and exercise self-efficacy scale scores (preintervention: mean 33.9, SD 4.5 points; postintervention: mean 35.9, SD 3.2 points; *P*=.043) were observed.

**Conclusions:**

The WheelFit app demonstrated promising feasibility, usability, and a positive impact on promoting PA in MWUs with SCI. Future investigation exploring the potential integration of the WheelFit app into clinical practice is warranted.

## Introduction

An estimated 302,000 people in the United States are living with spinal cord injury (SCI) as of 2023 [1]. On average, 39.3% of people in this population are manual wheelchair users (MWUs) [2]. In addition to direct changes in sensory and motor functions caused by the injury, 95% of people with SCI have at least one secondary complication [3]. Among these complications, obesity stands out with a prevalence of 66% [4,5]. A primary cause of obesity is the positive energy balance caused by decreased energy expenditure, often linked to reduced fat-free mass and physical activity (PA) in the SCI population [6]. Despite published PA guidelines, nearly half of this population remains sedentary and does not meet the minimum recommended guideline of 20 minutes of moderate to vigorous aerobic activity twice a week and 3 sets of 10 reps of strength training twice a week [7,8].

With the rapid development and cost reduction of commercial wearable devices, using such devices with mobile health (mHealth) apps to promote PA has become increasingly popular and shown promising benefits among the general population [9,10]. However, such success has yet to be fully realized among MWUs with SCI. One significant barrier is that the majority of commercial wearable devices are developed and optimized for ambulatory individuals, lacking validity for MWUs with SCI. As of November 2024, only the Apple Watch and certain Garmin smartwatches provide a wheelchair mode for push count assessment [11-13]. While the Apple Watch accurately tracks push counts for wheelchair users, it is costly and requires an iPhone, limiting its use among Android users and those with limited financial resources [14,15]. Validation studies on the Garmin smartwatch in the wheelchair mode are still lacking.

Currently, only a few studies have explored the use of commercial wearable devices in mHealth interventions aimed at promoting PA in MWUs with SCI. The majority of these studies mainly use wearable devices as outcome measurement tools. The Workout on Wheels internet intervention (WOWii) developed by Froehlich-Grobe et al [16] used a Polar A300 fitness tracker to manually log workout durations from 143 participants during workout sessions delivered through the WOWii web portal. The workout minutes were used for workout progress monitoring, which was displayed on the WOWii site to help participants track their progress toward established goals. The 24-week study comprised a 16-week intervention phase followed by an 8-week maintenance phase. During the intervention phase, 73% (123/168) of participants logged their workouts using the Polar fitness tracker, while approximately 29% (48/168) to 41% (69/168) continued to do so during the maintenance phase. Analysis of workout minutes revealed a significant increase in aerobic and strength workout time among participants during the intervention phase, a trend that was maintained through the maintenance phase [16].

Another mHealth intervention that used commercial wearable devices is the Scale-Up Project Evaluating Responsiveness to Home Exercise And Lifestyle Tele-Health (SUPER-HEALTH) developed by Rimmer et al [17]. The SUPER-HEALTH intervention is designed for individuals with physical disabilities, including those who can ambulate independently as well as those who use wheelchairs. Similar to the WOWii project, the SUPER-HEALTH delivered workout content through the website and used a Fitbit Charge fitness tracker to monitor participants’ PA data. The PA was displayed to the participant in the activity dashboard of the SUPER-HEALTH site, and the study personnel also monitored the PA data and manually issued reminders to maintain intervention fidelity. The full quantitative results of the SUPER-HEALTH study are not available yet. It is worth noting that the Fitbit devices were designed to measure PA in the general population, and their validity in people with SCI remains unknown. Using such PA data may lead to biased outcome measurement [18,19].

On the other hand, the Personal Health Informatics and Rehabilitation Engineering (PHIRE) app developed by Hiremath et al [20] is a wearable device-oriented mHealth PA intervention for MWUs with SCI. It provides PA measures using data from a commercial LG Android smartwatch and a wheel sensor, processed by custom algorithms for MWUs with SCI [20]. The app quantifies participants’ daily PA as energy expenditure, wheelchair travel distance, and PA minutes. The app was designed following the Just-in-time Adaptive Intervention (JITAI) framework, which aims to provide the right intervention at the right time [21]. Once the user completes a bout of PA, defined as a minimum of 3 continuous moderate to vigorous physical activity (MVPA) minutes, the app provides congratulations and issues JITAI messages based on various circumstances. If the user has not met their daily MVPA minutes goal, the app displays the current progress and the remaining minutes needed to reach the goal. Upon achieving the goal, the app congratulates the user. If the user exceeds the goal, the app shows the current accumulated MVPA minutes. Their pilot study results indicated that out of 16 participants, 38% (6/16) and 69% (9/16) of participants experienced a considerable increase (>10%) in light and moderate intensity PA, respectively [20]. While the PHIRE app integrates a wearable device with a validated algorithm for MWUs with SCI, it lacked the adaptive workout features found in the SUPER-HEALTH and WOWii studies.

Incorporating both wearable-based PA monitoring and mHealth-delivered adaptive workouts, we conducted this WheelFit study with 2 main aims. The first was to develop the WheelFit fitness app for MWUs with SCI. This app integrates daily PA monitoring and feedback using data from a commercial smartwatch and a wheelchair sensor, processed with custom algorithms for this population. Additionally, the app includes an adaptive workout library created by health care professionals. The second aim was to evaluate the feasibility and usability of WheelFit as an mHealth intervention for individuals with SCI.

## Methods

### WheelFit Devices

**Figure 1.**
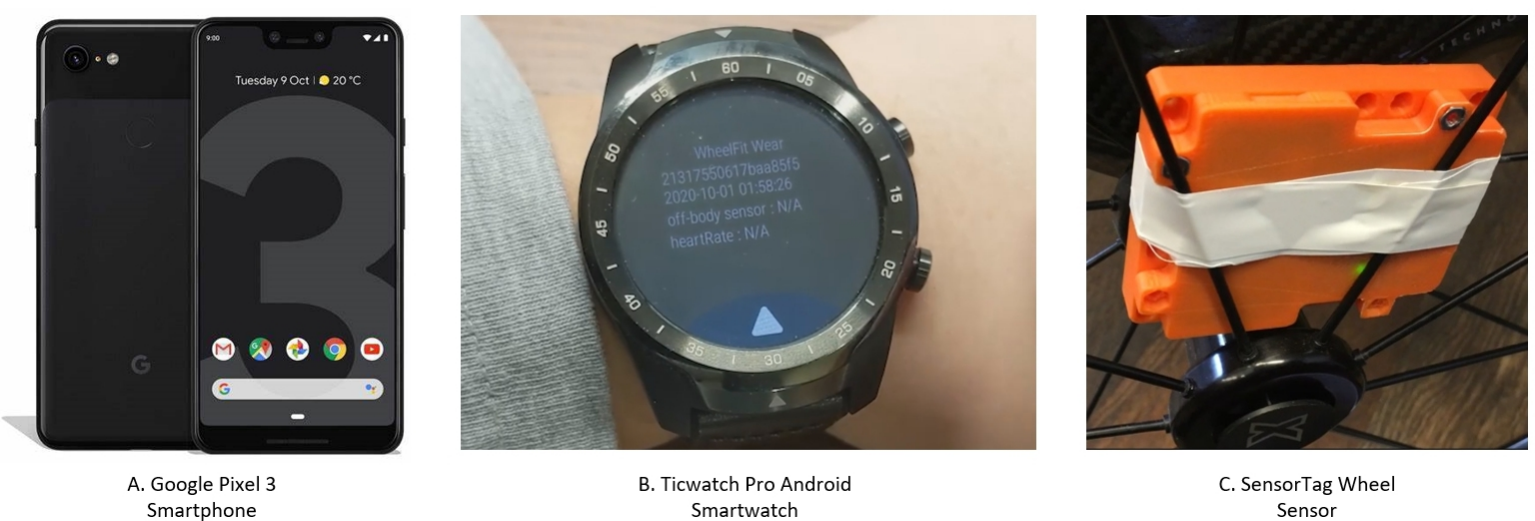
WheelFit devices. (A) Google pixel 3 smartphone, (B) Ticwatch Pro Android smartwatch, and (C) SensorTag wheel sensor.

The WheelFit app is designed for Android and is deployed to pixel 3 phones (Google LLC), as shown in Figure 1A. The app communicates with two wearable devices to calculate PA variables: a TicWatch Pro smartwatch (Mobvoi Information Technology LLC), shown in Figure 1B, and a SensorTag wheelchair sensor (Texas Instruments), shown in Figure 1C.

The TicWatch Pro is an Android WearOS-based smartwatch. We developed a WheelFit companion app for the smartwatch, which samples data from its accelerometer at 30 Hz and from the heart rate sensor at 1 Hz. Raw acceleration signal data are packed using the “batching” function of the WearOS and sent to the smartphone app each minute. The heart rate data are streamed to the smartphone app continuously. The companion app also shows the connection status and issues vibration notifications when the watch is disconnected from the phone or a sedentary behavior is detected. The battery of the smartwatch lasts about 10‐11 hours.

The SensorTag is a multisensor Internet-of-Things device [22]. We developed custom firmware that samples only the accelerometer and gyroscope, deactivating other sensors to conserve battery life. The accelerometer is used to activate the gyroscope when wheelchair movement is detected. The gyroscope registers the wheel rotation at 5 Hz. The device then converts the number of rotations to the distance traveled according to the wheel diameter specified in the settings and continuously streams all data to the smartphone app through Bluetooth Low Energy protocol. The SensorTag is attached to the wheelchair spokes using a custom 3D-printed case. Powered by 2 AAA batteries, it has a battery life of 1.5 months.

### WheelFit App Development

#### Conceptual Framework

We developed the WheelFit app based on the Fogg Behavioral Model (FBM) with inputs from stakeholders, including MWUs, physical therapists (PTs), and personal trainers, to promote PA in MWUs. The FBM includes three key factors: motivation, ability, and triggers [23]. It suggests that for a target behavior to occur, an individual must be sufficiently motivated, have the ability to perform the behavior, and be prompted by a trigger.

#### The WheelFit App

**Figure 2.**
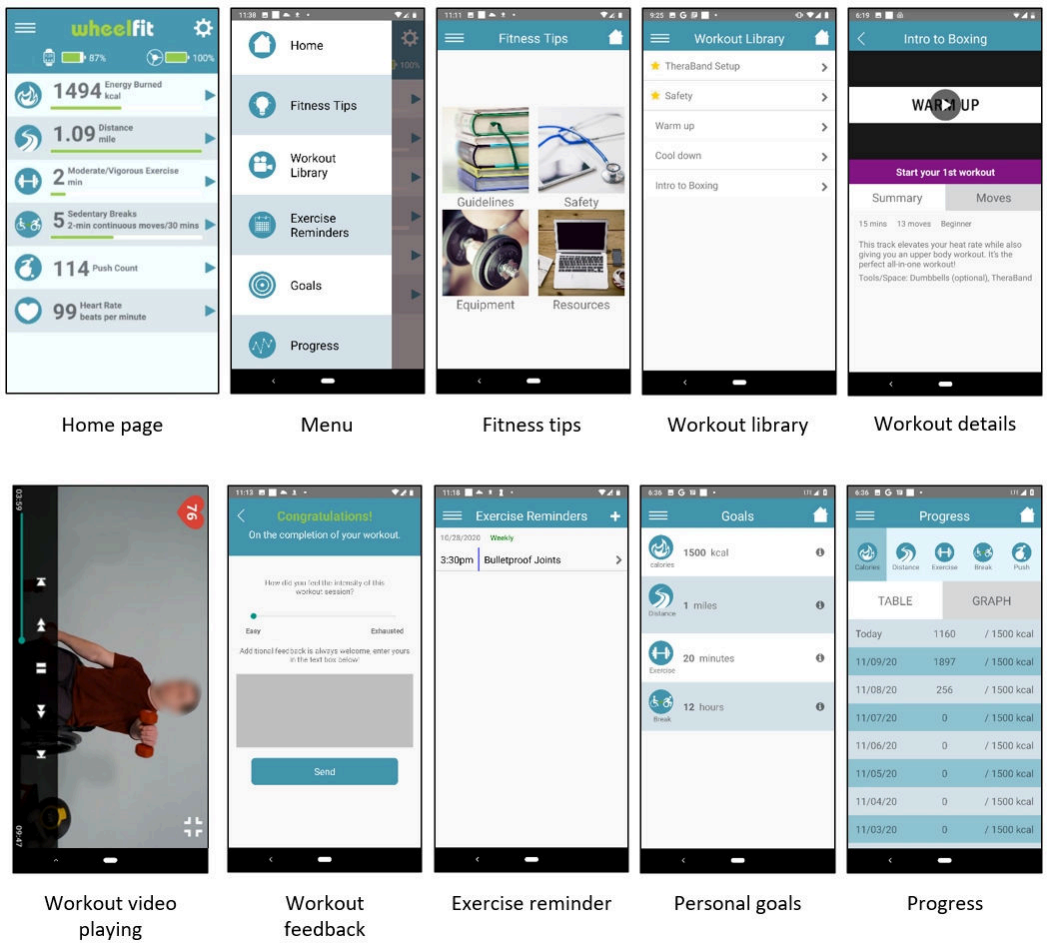
The WheelFit app.

The WheelFit app adopted the multicomponent design (Figure 2), which has proven to be more effective than the single-component design, to address the three FBM key factors [24].

To address ability, the FBM suggests that making desired behavior easier to perform is simpler than training individuals to have new skills [23]. The WheelFit app meets this requirement by offering an adaptive workout library with short, accessible workout videos designed to lower the knowledge and time barriers to PA, as identified in previous research [25].

The app incorporates triggers to prompt actions [23], using smartwatch push notification reminders and vibration alerts for sedentary behavior. These triggers encourage users to engage in both structured and unstructured PA, ensuring consistent engagement and adherence to PA routines.

To enhance motivation, the app includes daily PA monitoring, fitness tips, goal setting, and progress-monitoring modules. The daily PA-monitoring module, featured on the app’s home page, offers objective monitoring to support self-regulation, while the fitness tips module provides educational content on the benefits of PA. The goals and progress modules further motivate users by highlighting their achievements and monitoring their PA history [26,27].

#### Home Page (Daily PA Monitoring)

The home page layout includes a menu icon, a settings icon, device connection indicators for the watch and wheel sensor, and six daily PA measures. Users can navigate through the settings icon to the settings page to enter or update their demographic information and adjust the wheelchair wheel diameter for a more precise PA assessment. The device connection indicator shows the battery level of each connected sensor and displays the connection status. The six PA measures are (1) energy burned, (2) distance traveled, (3) moderate to vigorous exercise time, (4) sedentary breaks, (5) total push counts, and (6) real-time heart rate. The energy burned is estimated using a custom prediction model originally developed for ActiGraph devices in our previous studies and adapted to the Android platform [25,28]. The distance traveled is the cumulative distance calculated from the number of rotations by the wheelchair sensor. For the moderate to vigorous exercise time, the app first calculates the mean absolute deviation from the accelerometer data of the current minute and then applies the thresholds established by Shwetar et al [29] to classify the activity intensity into sedentary, light, or MVPA. The accumulated MVPA minutes are shown as moderate to vigorous exercise time. The sedentary break is defined as the total number of 30-minute periods where at least 2 consecutive minutes of activity above the light intensity are registered. For example, if a user is awake for 16 hours, the maximum possible number of sedentary breaks per day is 32. If the app detects that a sedentary break has not occurred within the initial 25 minutes of a 30-minute interval, it triggers a sedentary behavior alert through vibrations and texts on the smartwatch. The total push count shows the cumulative number of wheelchair pushes calculated through the application of algorithm by Veerubhotla [30] using the raw accelerometer data collected from the smartwatch. Finally, the real-time heart rate variable shows the most current measurement from the smartwatch’s heart rate sensor.

#### Fitness Tips Page

The fitness tips page contains educational materials we have compiled regarding current PA guidelines, wheelchair exercise safety, exercise equipment and tools, and related resources, all formatted as web papers.

#### Workout Library Page

We provide an adaptive workout library containing 11 workout tracks in three categories: (1) aerobic (3 tracks), (2) strengthening (5 tracks), and (3) flexibility exercises (3 tracks). Each track, lasting approximately 15 minutes, comprises a warm-up, several brief movements (around 1‐2 minutes each), and a cooldown. These workouts are developed by clinicians and feature wheelchair users, drawing on research evidence and incorporating feedback from users. The description page of each workout track provides details such as workout duration, the number of movements, difficulty level, required equipment or space, and explanations. Given the diverse physical abilities and impairments of individuals with SCI, certain movements may be appropriate for some users but could present risks to others who have reduced range of motion or muscle strength. Therefore, we introduced the workout edit function, enabling health care providers or personal trainers to categorize each movement as “safe to perform,” “adapt with modification,” or “skip movement” while also offering the ability to highlight or hide each workout. When playing the workout videos, users can use the media control to start, pause, fast forward, and rewind the video. Users’ real-time heart rate is displayed at the top left corner to provide an estimation of workout intensity. Upon completion of a workout, the app will prompt the user to rate their perceived exertion and share any feedback they may have.

#### Exercise Reminders Page

The exercise reminders page provides functions for users to create, edit, and delete exercise reminders. Users can designate the start time, workout type, recurrence, and alarm for the reminders. The app syncs the reminders with the Google Calendar app on the phone to use its built-in notification functions.

#### Goals Page

The goals page allows users to set their daily goals for energy burned, distance traveled, moderate to vigorous exercise minutes, and sedentary breaks. Goals are also reflected as progress bars on the home page. Users can view the progress bars to track daily progress toward goals.

#### Progress Page

The progress page presents historical daily PA variables except for the heart rate. Users can review their past data with either a table view that shows detailed numbers or a graph view that visualizes the trends of the past 30 days.

### Feasibility and Usability Evaluation

#### Participants

Participants were included in the study if they were (1) aged between 18 and 65 years, (2) diagnosed with an SCI, (3) at least 1-year postinjury (if acquired) and medically stable, (4) not consistently engaging in regular PA for at least 6 months, (5) using a manual wheelchair as their primary means of mobility, (6) living in a community, and (7) experienced in using a smartphone. Potential participants were excluded if they had any active pelvic or thigh wounds or were pregnant (based on self-report). They were also screened for medical conditions (eg, cardiovascular diseases, stroke, pressure ulcers, fracture, musculoskeletal injuries, or COVID) that could limit PA participation. This screening involved a series of questions regarding hospitalizations and therapy received during the past year, physician clearance, and self-perceived limitations as related to each condition identified. If any potential conditions were identified, our PT reviewed the responses, conducted necessary follow-up, and determined whether the participant could safely participate, required further clearance from their primary care provider, or should be excluded. Participants were recruited by convenience sampling through study registries, flyer distribution, and direct outreach.

Power analysis was conducted based on PA effectiveness measurement (ie, daily moderate to vigorous exercise minutes) using data from a mHealth study on adults with diabetes aimed at improving daily light activities (Cohen *d*=0.93), as no relevant literature on mHealth interventions for individuals with SCI was available [31]. Assuming a power of 0.8 and an α level of .05, G*Power (Heinrich-Heine-Universität Düsseldorf) indicated a required sample size of 13 [32]. To account for a potential 20% attrition and compliance rate, we planned to recruit 16 participants.

#### Study Design

**Figure 3.**
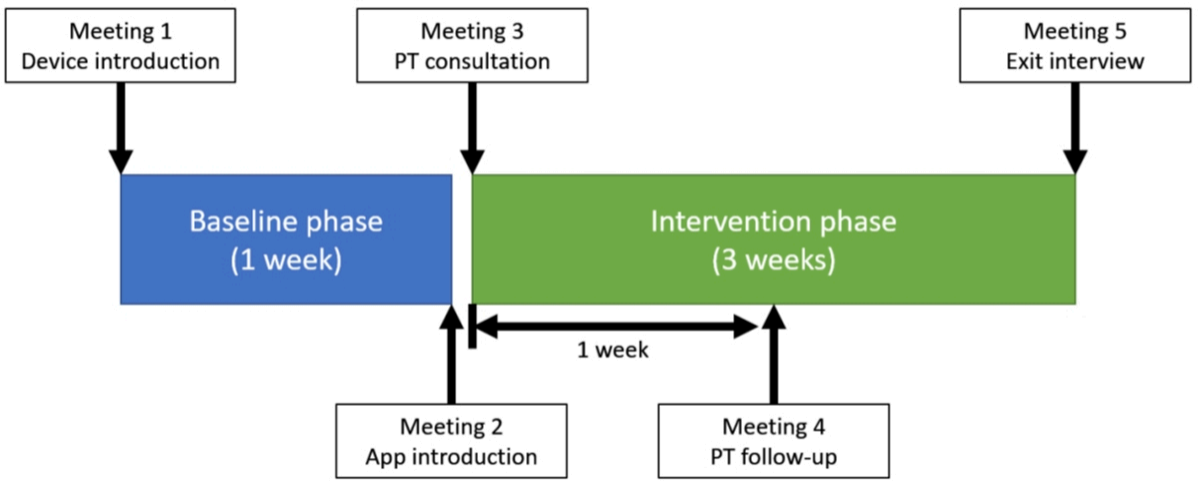
Study procedure. PT: physical therapist.

The 4-week full-remote study consisted of a 1-week baseline phase and a 3-week intervention phase, with 5 virtual meetings held via Zoom (Zoom Video Communications, Inc) (Figure 3). After informed consent, participants completed the demographics and SCI exercise self-efficacy scale (ESES) questionnaire via REDCap (REDCap Consortium) [33]. A study package was mailed to participants that contained a Google Pixel 3 smartphone, a TicWatch Pro smartwatch, a Samsung Galaxy tablet, a SensorTag wheel sensor, two sets of TheraBands, a wheelchair pouch, a tablet stand, a universal charger, and written instructions. Upon receiving the study package, the participant had the first meeting with the researchers. During this first virtual meeting, a researcher provided instructions on how to use the devices, with the participant following along using the study devices. The researcher also addressed any questions raised. Then, the participant was asked to independently complete a series of tasks to ensure their proficiency in using the devices. In case of any unsuccessful attempts, additional explanations and instructions were provided.

##### Baseline Phase

The 7-day baseline phase began after the first meeting concluded. To maintain the integrity of participants’ typical PA, most functions of the WheelFit app, including the home screen PA display and workout library, were deactivated. Participants were instructed to maintain the connection between all devices so that the app could record the PA silently. The participants were also asked to complete a daily log to record their bedtime and waketime and indicate whether each day was a typical day, defined as a day with routine activities without any unexpected events that affected their PA level (eg, illness). The daily log was sent to the participants’ email every day throughout the study.

At the end of the baseline phase, participants were scheduled for a second meeting to introduce the WheelFit app. During this meeting, a researcher activated all functions of the WheelFit app and provided corresponding instructions. Participants were then asked to perform a series of tasks independently to demonstrate their understanding of the app. If participants were unable to complete a task, additional guidance was provided.

### Intervention Phase

The day after the second meeting, participants had the third meeting with our licensed PT and personal trainer, both of whom had experience working with people with physical disabilities. Including both professionals ensured the safety of our study and provided flexibility in scheduling follow-up sessions. In the third meeting, our PT assessed the participant’s knowledge of PA and gathered self-reported information, including wheelchair details, assistive technology use, medical and injury history, living situation, and transfer techniques. A functional assessment was conducted, covering the range of motion, muscle strength, and sensation. Due to the remote nature of the meeting, the PT visually assessed the range of motion via Zoom, guiding participants through various postures. Muscle strength and sensation information was collected based on participants’ self-report. Traditional aerobic capacity assessments, which require emergency assistance personnel in laboratory settings, were not feasible in our remote format [34]. After the functional assessment, our personal trainer provided an educational session on PA, sedentary behavior, and the specific, measurable, achievable, relevant, and time-bound goal setting. The PT and personal trainer also assisted the participants in making an action plan for engaging in workouts according to the participant’s functional assessments and personal goals. The PT and personal trainer directed the participants to perform all movements in each prescribed workout while observing their performance via Zoom. They provided feedback to correct improperly performed movements and suggested alternatives for movements that did not align with the participant’s functional ability. If no alternative was available, the participant was instructed to skip the movement. All alternative or skipped recommendations were recorded in the WheelFit app. The completion of the third meeting marked the beginning of the 3-week intervention phase.

Participants followed their action plans using the WheelFit app for 1 week. Then, they had the fourth meeting held with the PT or personal trainer to address any questions and adjust the action plan as needed. Participants were instructed to adhere to their updated action plans for the remainder of the intervention phase. At the end of the intervention phase, participants had the fifth meeting with a researcher, where they completed the system usability scale (SUS) for WheelFit, the postintervention ESES, and a participant perception of use survey.

### Outcome Measures

#### PA Measures

Since the goal of the WheelFit app is to promote PA and reduce sedentary behavior in MWUs with SCI, we consider “moderate to vigorous exercise time” and “sedentary breaks” as the primary outcomes for PA. The rest of the PA variables were not selected as they were not appropriate for goal setting and were only for participants’ references. All calculated PA measures were stored on the study smartphone and retrieved after the participants mailed back the devices.

#### Exercise Self-Efficacy

Participants’ exercise self-efficacy was assessed using the SCI ESES, a 10-item, 4-point Likert scale assessing an individual’s confidence toward PA [33]. This scale has been validated in individuals with SCI [33]. The possible scores range from 10 to 40, with a higher score indicating higher confidence toward PA. The ESES was assessed before and after the study using REDCap questionnaires.

#### Intervention Feasibility

The feasibility of the WheelFit intervention was evaluated in three areas: (1) session attendance, (2) device and app usage, and (3) action plan implementation. The session attendance was assessed as attendance of all study meetings except for the exit meeting, which is not considered part of the WheelFit intervention. If a participant missed a meeting but attended a rescheduled session, it was counted as valid attendance. Conversely, if a participant missed a meeting and was unwilling to attend any rescheduled sessions, it was marked as not attended. The device and app usage were assessed as the time participants maintained a connection between the smartwatch and the smartphone. The implementation of the action plan was assessed based on the completion rate of the prescribed workouts in each participant’s action plan.

#### WheelFit App Usability

The usability of the WheelFit app was assessed through the SUS. The SUS is a 10-item, 5-point Likert scale that assesses users’ perceived usability of the app. The possible scores range from 20 to 100. An SUS score larger than 80.3 indicates excellent app usability, while a score between 68 and 80.3 indicates that the app has acceptable usability but there is a need for improvement. A score below 68 indicates that the app has poor usability and needs to be revised [35-37]. The SUS has been validated and widely used to assess the usability of mHealth apps [35,38-41].

The participant feedback was evaluated through a participant perception of use survey. The survey included three questions: (1) a 4-point Likert scale question regarding the efficacy of WheelFit in facilitating an active lifestyle (ie, very helpful/somewhat helpful/not very helpful/not at all helpful), (2) a 4-point Likert scale regarding participants’ perception of any health or functional improvements (ie, to a great extent/somewhat/very little/not at all), and (3) a question regarding participants’ willingness to recommend WheelFit to other MWUs (ie, yes/no/cannot decide).

### Data Preparation and Analysis

After retrieving PA data from the study phone, valid day data were extracted. A day is considered valid if it meets the following three criteria: (1) the daily data file must contain at least 30% of nonnull or nonzero smartwatch accelerometer data (approximately 8 hours, which is the minimum estimated smartwatch battery life after 1 full charge), (2) no abnormal data were observed (eg, zero wheelchair pushing distance), and (3) the participant confirmed in the daily log that the day was a typical day, with no events altering their daily routine. All other data were retrieved from REDCap.

The demographic data were analyzed at the group level. All valid day PA data were aggregated and averaged to the daily level for the baseline and intervention phase, respectively. Each participant’s preintervention and postintervention PA data were presented in a table for a more detailed interpretation. The overall differences between PA variables (ie, moderate to vigorous exercise time, sedentary break, and travel distance) among all participants during the 2 phases and the ESES scores before and after the intervention were compared using a 2-tailed paired sample *t* test or its nonparametric equivalent if assumptions were not met. To further analyze individual PA change, we defined a ±10% change as the threshold for a “considerable” level of change for the “moderate to vigorous exercise time” based on prior studies [20]. Brief descriptions of participants’ PA background, personal goals, and motivations were presented to aid in contextualizing the change in PA with the use of the app during post hoc analysis. The action plan workout prescription and execution were also presented. For feasibility and usability evaluation, the session attendance and device connection rate were reported. Descriptive analysis was used to summarize the SUS scores of WheelFit and participants’ responses to the participant perception of use survey. Participants’ digital literacy was assessed through observations of their interactions with the study devices and the WheelFit app, as well as relative information shared during all study meetings. This evaluation of their digital literacy level was used to enhance the comprehension of app usability during post hoc analysis. All statistical analyses were conducted using SPSS (version 28; IBM Corp), Microsoft Excel (Microsoft Corp), and Python (version 3.0; Python Software Foundation).

### Ethical Considerations

This study was approved by the institutional review board at the University of Pittsburgh (STUDY19040049). Informed consent was obtained from all participants, who were also reminded of their right to withdraw from the study at any time. Identifiable data, such as meeting recordings, were securely stored on a university-approved drive with access restricted to the core research team. All data were deidentified prior to analysis to ensure participant confidentiality. Participants received a total compensation of US $225 for full participation in the study.

## Results

**Figure 4.**
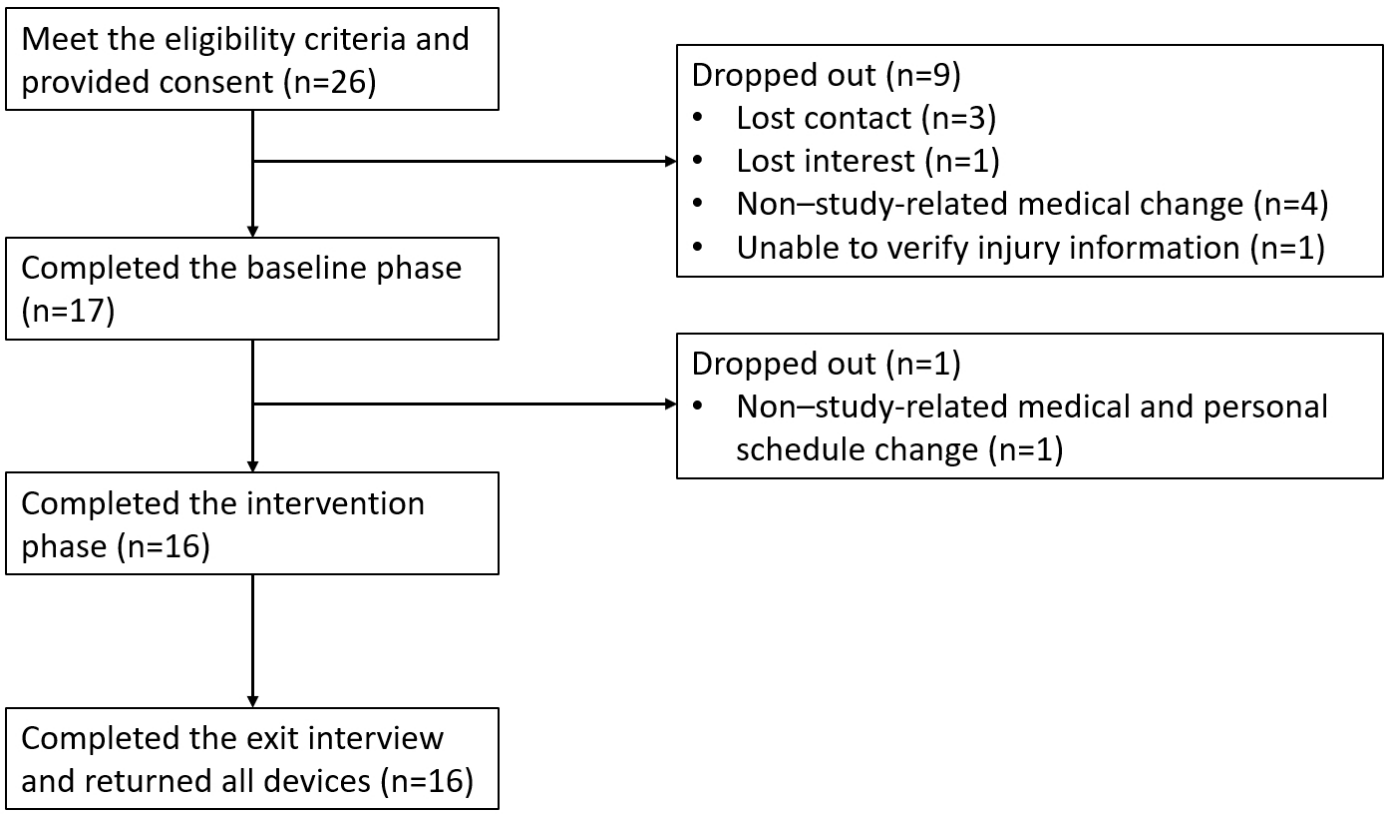
Study participant diagram.

The study commenced in November 2020 and ended in October 2022. A total of 26 participants enrolled in the study, with 16 participants completing the full study protocol. Of the 10 withdrawn participants, 9 dropped out before the study began (meeting 1) and 1 dropped out during the intervention phase. All dropouts are non–study-related. Figure 4 shows the participant diagram.

The demographics of the 16 participants who completed the study are provided in Table 1.

**Table 1.** Demographics.

Demographics variable	Participants
Age (years), mean (SD)	48.0 (9.8)
Height (cm), mean (SD)	173.6 (10.2)
Weight (kg), mean (SD)	75.2 (18.6)
BMI, n (%)	
≤25	7 (43.8)
25 < BMI <30	5 (31.3)
≥30	4 (25.0)
Sex, n (%)	
Male	8 (50)
Female	8 (50)
Ethnic origin, n (%)	
Caucasian	13 (81.3)
African American	3 (18.8)
Injury type, n (%)	
SCI: complete^a^	5 (31.3)
SCI: incomplete	7 (43.8)
Spinal cord disease	1 (6.3)
Not reported	3 (18.8)
SCI level, n (%)	
C1-C8^b^	2 (12.5)
T1-T12^c^	9 (56.3)
L1-L5^d^	1 (6.25)
Not reported	4 (25.0)
Self-rate fitness level, n (%)	
Very good	4 (25.0)
Good	6 (37.5)
Fair	6 (37.5)
Self-rate nutritional habits, n (%)	
Excellent	1 (6.3)
Very good	4 (25.0)
Good	9 (56.3)
Fair	2 (12.5)
Employment status, n (%)	
Employed	6 (37.5)
Unemployed	10 (62.5)

a SCI: spinal cord injury.

b C: cervical.

c T: thoracic.

d L: lumbar.

Table 2 shows the average daily PA levels of the 16 participants during the baseline and intervention phases, along with the corresponding percentage change. The Wilcoxon signed rank tests indicated that there is a significant increase in moderate to vigorous exercise time during the intervention phase (median= 28.4) compared with the baseline phase (median=26.9, *Z*=1.97; *P*=.049; *r*=0.49), but no significant change in the sedentary break was found (*Z*=0.52; *P*=.605; *r*=0.13). Two-tailed paired sample *t* tests revealed that participants’ ESES scores were significantly higher after the intervention (mean 35.9, SD 3.2) than before the study began (mean 33.9, SD 4.5; *t*_15_=1.84; *P*=.043; *d*=4.35). A total of 9 participants (ie, participant numbers 2, 3, 4, 5, 8, 15, 16, 17, and 19) had a considerable increase in exercise time, 2 participants (ie, participant numbers 7 and 10) had a considerable decrease, while the remaining 5 participants (ie, participant numbers 11, 14, 21, 25, and 26) had no considerable change.

**Table 2.** Changes in physical activity outcomes.

ID	Moderate to vigorous exercise time (minutes)		Sedentary break (counts)		Travel distance (miles)		Exercise Self-Efficacy Scale (score)	
	Baseline	Intervention, change(%)	Baseline	Intervention, change(%)	Baseline	Intervention, change(%)	Baseline	Intervention, change(%)
02	2.8	4.4 (57)	14.8	13.5 (−9)	0.4	0.4 (0)	37.0	37.0 (0)
03	27.9	37.0 (33)	15.4	19.1 (24)	0.6	0.6 (0)	32.0	35.0 (9)
04	26.8	99.0 (269)	9.4	12.3 (31)	0.1	0.2 (100)	40.0	39.0 (−3)
05	42.9	73.3 (71)	19.4	20.1 (4)	0.7	1.3 (86)	34.0	38.0 (12)
07	47.0	17.1 (−64)	24.0	13.8 (−43)	1.7	0.8 (−53)	34.0	34.0 (0)
08	23.9	27.8 (16)	17.2	17.8 (3)	0.5	0.6 (20)	31.0	30.0 (−3)
10	57.8	44.1 (−24)	17.6	18.1 (3)	1.0	1.0 (0)	40.0	39.0 (−3)
11	45.0	48.5 (8)	22.0	21.7 (−1)	1.4	0.8 (−43)	37.0	40.0 (8)
14	11.8	11.1 (−6)	14.1	11.8 (−16)	1.0	0.7 (−30)	27.0	34.0 (26)
15	18.6	21.5 (16)	18.2	17.7 (−3)	2.1	1.8 (−14)	27.0	36.0 (33)
16	1.1	9.9 (800)	13.2	11.9 (−10)	0.2	0.4 (100)	33.0	34.0 (3)
17	32.9	39.6 (20)	20.4	20.9 (2)	0.6	0.8 (33)	30.0	37.0 (23)
19	5.9	18.1 (207)	13.0	17.9 (38)	0.4	0.8 (100)	38.0	37.0 (−3)
21	27.0	29.0 (7)	19.4	16.1 (−17)	0.8	0.7 (−13)	39.0	39.0 (0)
25	12.9	13.5 (5)	15.1	13.9 (−8)	0.1	0.3 (200)	36.0	29.0 (−19)
26	38.8	39.3 (1)	17.6	16.5 (−6)	1.1	0.8 (−27)	28.0	37.0 (32)
Mean (SD)	26.4 (16.9)	33.3 (24.9)	16.9 (3.7)	16.4 (3.2)	0.8 (0.6)	0.7 (0.4)	33.9 (4.5)	35.9 (3.2)

Most participants were prescribed 2-3 workouts per week based on their functional assessments and personal goals. Strength training workouts were prescribed to 14 participants, aerobic workouts to 9 participants, and flexibility workouts to 9 participants. Among the 16 participants, 4 participants consistently adhered to or exceeded their workout action plan for all 3 intervention weeks. Three participants followed their action plan for 2 weeks but missed some workouts during 1 week. Four participants adhered to their prescriptions for 1 week, while the remaining participants practiced the workout occasionally but did not consistently follow the action plan throughout the intervention phase.

In terms of feasibility, all 16 participants had 100% attendance at all study meetings. On average, they maintained the connection between the smartwatch and the WheelFit app for about 59% of the time (14.2 hours) every day. This wear time satisfies the daily valid wear threshold of 10 hours per day, which has been commonly adopted in wearable device–based PA studies [42].

An average SUS score of 81.8 (SD 19.2) was found. Four participants (ie, nos. 2, 4, 10, and 16) reported an SUS score under the “poor” usability threshold of 68. In terms of the participant perception of use survey, all participants reported that the intervention was helpful in facilitating an active lifestyle, with 11 participants rating it as “very helpful,” 4 rating it as “somewhat helpful,” and 1 rating it as “helpful.” For the subjective perception of health and function improvement, a total of 8 participants reported a “to a great extent,” 6 participants reported “somewhat,” and 2 participants reported “not at all.” All participants expressed that they would recommend WheelFit to others.

## Discussion

### Principal Findings

In this study, we developed the WheelFit mHealth app to promote PA in MWUs with SCI. Following the FBM, we integrated wearable device-based PA monitoring to increase users’ motivation, an adaptive workout library to enhance their abilities, and wearable push notifications and vibration alerts to trigger users to engage in PA and break sedentary behavior. We further evaluated the feasibility and usability of a PA intervention facilitated by WheelFit in MWUs with SCI through a 4-week single-group pre-post study. A total of 16 participants completed the study protocol with a 100% session attendance rate. Significant improvements in PA outcomes and exercise self-efficacy were observed. The WheelFit app also received a high SUS score of 81.8, with all participants reporting favorable subjective perceptions regarding the intervention. The WheelFit appears to be a feasible and potentially effective mHealth intervention for promoting PA in MWUs with SCI.

### Comparison With Prior Work

In terms of the WheelFit intervention’s impact on participants’ PA, our results showed a statistically significant increase in average MVPA (exercise time) minutes, indicating a positive effect on exercise time among participants.

These findings align with trends observed in other mHealth interventions for MWUs with SCI. Among these interventions, the PHIRE app study is most comparable with ours, as it also used wearable devices. However, it uses a different behavior intervention strategy based on the JITAI framework. Despite the difference in some behavioral components as well as the participants’ age (WheelFit: mean 48.0, SD 9.8 years; PHIRE: mean 29.3, SD 28.1 minutes), the baseline MVPA (WheelFit: mean 26.4, SD 16.9 minutes; PHIRE: mean 29.3, SD 28.1 minutes) were similar [20]. Notably, in both studies, 56% (9/16) of participants experienced a considerable (>10%) increase in MVPA minutes [20]. The WOWii study involved participants of similar age (mean 48.0, SD 9.8 years). Its virtual workout intervention, accompanied by support linkage (eg, behavior skill promotion and peer support), focused on promoting structured exercises [16]. Over the 16-week intervention phase, participants successfully increased their aerobic exercise minutes from less than 100 minutes per week to more than 150 minutes per week and their strength training frequency from less than 3 days per week to more than 3.5 days per week. During the 8-week maintenance phase, participants maintained aerobic exercise levels above 150 minutes per week and achieved an average of 3.3 days per week of strength training [16].

After a closer look into participants who had a considerable increase in exercise time, we noticed that participant numbers 2, 16, and 19 had very low baseline PA, and all expressed a desire to use the WheelFit app to form a more physically active lifestyle. Participant numbers 4 and 17 in this group reported prior experience with wheelchair sports and tried to use the WheelFit app to enhance their PA training for the upcoming games. Participant numbers 5 and 8 reported that they were driven by a commitment to maintain good health and enhance body shape. Participant numbers 3 and 15 did not report any significant motivators and simply followed the recommendations of our PT and personal trainer. For the 2 participants who had a considerable decrease in exercise time, participant number 7 reported experiencing severe chronic neurological pain that prevented this participant from engaging in PA and any of the study workouts. Chronic pain has been identified as one of the barriers to daily PA by multiple studies, including the PHIRE study [20,43,44]. Therefore, pain management is an essential factor that should be considered in future mHealth studies. Participant number 10 reported recently starting a daily routine of visiting the local gym and opted not to engage in the prescribed workouts, deeming them too easy. The significant decrease in this participant’s PA amount was presumably due to the high baseline PA status and normal fluctuation in the PA routine. Of the remaining 5 participants, 4 of whom experienced a slight increase in exercise time, and 1 experienced a minor decrease.

The participants in this study have varied preparticipation PA levels. Some participants had experience in wheelchair sports, while others were novices to structured training. Additionally, some participants had limited knowledge of PA and led a highly sedentary lifestyle. During the COVID-19 pandemic, MWUs with SCI had reduced access to health care providers and decreased PA amount due to the closure of facilities and concern about infection [45]. Under this circumstance, the WheelFit intervention may benefit people with SCI in 2 ways. For those who were actively seeking engagement in PA, the WheelFit app provided a new approach to increasing PA at home and potentially alleviating the pandemic’s negative impact. Participant numbers 4 and 17 were the representative cases. Such benefit may also be generalized to potential users whose PA lifestyle is affected by factors such as cold weather and geographic location [46]. On the other hand, participants who lived a sedentary lifestyle experienced less impact from the pandemic as their baseline PA was already low. The WheelFit mHealth intervention assisted these participants in starting to engage in more PA by providing PA knowledge through consultation and PA monitoring through the app. Such a trend is further supported by participants’ feedback, indicating that the WheelFit app facilitated them to adopt a physically healthier lifestyle.

Overall, the statistically significant increases in daily exercise time suggest that the use of WheelFit increased participants’ PA levels. In addition, the statistically significant increase in ESES scores indicates that the participants became more confident in engaging in PA. As anticipated, there was no increase in wheelchair travel distance as our PT and personal trainer advised the participants not to use travel distance as an indicator of PA level and did not use this variable to compose their action plan. More specifically, excessive wheelchair pushing may increase the risk of shoulder injury instead of bringing health benefits [47]. Regarding the sedentary breaks, we noticed that many participants had a high number of breaks that were approaching the smartwatch’s battery life, indicating a ceiling effect that highlights the limitation of our current definition of sedentary breaks—2 consecutive minutes of nonsedentary PA. Currently, in SCI research, there is no consensus on the definition of sedentary behavior or the ideal bout length [48]. It has been found that breaking up sedentary bouts to under 20 minutes with at least 1 minute of nonsedentary PA could yield health benefits [49]. Validating such effects could be valuable in the SCI population as it informs the development of future mHealth interventions. Strength training was the most commonly prescribed workout, with 14 out of 16 participants selecting it as a goal due to their limited knowledge and access to appropriate resources. Notably, flexibility training was prescribed to 9 out of 16 participants—the same as aerobic training—highlighting a strong interest in addressing joint tightness and muscle pain. Despite its importance, flexibility training is often overlooked in SCI exercise guidelines, which primarily emphasize aerobic and strength training [7,44]. Given its role in managing muscle stiffness and pain and preventing injury, future PA studies and mHealth interventions should integrate flexibility training as a key component for this population [50].

In terms of usability, the high SUS score of 81.8 suggests that the WheelFit app had excellent usability. This is further supported by the participants’ overall positive feedback regarding the app. However, the large SD of 19.2 indicates that there is a large variance in participants’ perceived usability. Upon reviewing the meeting recordings and tech support records, it was found that participant numbers 2, 4, and 10 exhibited low digital literacy, a factor that has been found to negatively affect the usability of mHealth apps [51]. All 3 individuals experienced challenges in using the study smartphone and needed more assistance than others. Participant number 16, despite having good digital literacy, expressed frustration with the requirement to launch the WheelFit companion app on the smartwatch to maintain the connection. The participant also found it difficult to understand and remember the meanings of the PA variables displayed on the home page. In future studies, the assessment of digital and health literacy could be integrated into the mHealth intervention to investigate their effect on the intervention and provide personalized assistance in the MWU with the SCI population.

Given the promising outcomes, PTs can integrate WheelFit into rehabilitation by introducing adaptive workouts during sessions and empowering clients to use the app to perform and track their at-home exercises. Personal trainers working with MWUs with SCI can also leverage WheelFit to guide clients in establishing and maintaining a structured exercise routine. Additionally, WheelFit serves as a valuable self-guided tool, enabling users to independently engage in PA and adopt a more active lifestyle.

### Limitations

This study was conducted during the COVID-19 pandemic. Similar to other studies during this time period, factors such as the city lockdown and the limitation of health care resources caused the high non–study-related dropout rate of our study [52]. Given its pilot nature, this study used a single-group pre-post design. Without a control group, the study is unable to draw a definitive conclusion regarding the efficacy of the WheelFit intervention on PA. The intervention comprises multiple components, such as the wearable device, WheelFit app, and personal trainer, making it challenging to discern the essential and necessary elements. The 4-week duration is relatively short compared with other mHealth intervention studies, which can range from 6 to 48 weeks. For the ESES score improvement, as the minimal clinically important difference was not established for ESES, the clinical meaningfulness of such improvement remained unknown. It is worth noting that our participants exhibited a relatively high baseline ESES score (mean 33.9, SD 4.5 points), similar to the score for “exercisers” (mean 33.7, SD 5.3 points) reported in the original ESES manuscript [33]. This is probably due to our convenience sampling approach. As a result, the efficacy of the WheelFit mHealth intervention on MWUs with SCI who live a very sedentary lifestyle remained unknown. To ensure compatibility, we provided participants with a dedicated Android study smartphone, potentially affecting their interaction with the WheelFit app compared with using the app on their personal devices. With the WheelFit companion app running continuously, the smartwatch lasts only about 10‐11 hours before needing to be charged in the middle of the day. This limitation increased participants’ burden and was the main reason for daily PA data loss. Currently, the WheelFit app connects only with 1 wheel sensor, which lacks a quick attach and detach feature, potentially limiting its ability to track wheelchair movements for individuals using multiple wheelchairs, such as a sports or backup wheelchair. Future studies should contemplate a large-scale, multistage randomized design to delve deeper into the efficacy of the WheelFit intervention and the individual contributions of its components. Exploring the use of validated tools to assess participants’ digital and health literacy could offer a more nuanced understanding of its impact on app usage. Addressing pain management is also critical when developing PA interventions. Additionally, investigating strategies to enhance participants’ adherence to their action plans is also valuable.

### Conclusions

In this study, we developed WheelFit, a fitness app accompanied by wearable devices tailored for MWUs with SCI. The app offers population-specific PA monitoring with custom algorithms for MWUs with SCI, allowing these individuals to track their daily PA progress toward self-designated personal goals and review their PA history. It also provides an adaptive workout library, facilitating easy access to exercise options. We designed a pilot 4-week intervention using the WheelFit app, led by a PT and a personal trainer. The WheelFit app and intervention have shown promising feasibility and usability, suggesting the potential to promote PA in this population. Improvements in the app and device usability are essential for future development iterations.

## Abbreviations

ESES: Exercise Self-Efficacy Scale
FBM: Fogg Behavioral Model
JITAI: Just-in-time Adaptive Intervention
mHealth: mobile health
MVPA: moderate to vigorous physical activity
MWUs: manual wheelchair users
PA: physical activity
PHIRE: Personal Health Informatics and Rehabilitation Engineering
PT: physical therapists
SCI: spinal cord injury
SUPER-HEALTH: Scale-Up Project Evaluating Responsiveness to Home Exercise And Lifestyle Tele-Health
SUS: System Usability Scale
WOWii: Workout on Wheels internet intervention

## References

[1] (2020). Spinal cord injury facts and figures at a glance. National Spinal Cord Injury Statistical Center.

[2] SCIMS 2022 annual report—complete public version. National Spinal Cord Injury Statistical Center.

[3] Anson CA, Shepherd C (1996). Incidence of secondary complications in spinal cord injury. Int J Rehabil Res.

[4] Rajan S, McNeely MJ, Warms C, Goldstein B (2008). Clinical assessment and management of obesity in individuals with spinal cord injury: a review. J Spinal Cord Med.

[5] Farkas GJ, Sneij A, Gater DR (2021). Energy expenditure following spinal cord injury: a delicate balance. Top Spinal Cord Inj Rehabil.

[6] Nightingale TE, Williams S, Thompson D, Bilzon JLJ (2017). Energy balance components in persons with paraplegia: daily variation and appropriate measurement duration. Int J Behav Nutr Phys Act.

[7] Martin Ginis KA, Latimer-Cheung AE (2018). Evidence-Based Scientific Exercise Guidelines for Adults With Spinal Cord Injury: An Update and a New Guideline.

[8] Hwang EJ, Groves MD, Sanchez JN, Hudson CE, Jao RG, Kroll ME (2016). Barriers to leisure-time physical activities in individuals with spinal cord injury. Occup Ther Health Care.

[9] Liu JYW, Kor PPK, Chan CPY, Kwan RYC, Cheung DSK (2020). The effectiveness of A wearable activity tracker (WAT)-based intervention to improve physical activity levels in sedentary older adults: a systematic review and meta-analysis. Arch Gerontol Geriatr.

[10] Wang W, Cheng J, Song W, Shen Y (2022). The effectiveness of wearable devices as physical activity interventions for preventing and treating obesity in children and adolescents: systematic review and meta-analysis. JMIR Mhealth Uhealth.

[11] Karinharju KS, Boughey AM, Tweedy SM, Clanchy KM, Trost SG, Gomersall SR (2021). Validity of the Apple Watch^®^ for monitoring push counts in people using manual wheelchairs. J Spinal Cord Med.

[12] Apple Support. Use accessibility features on your Apple Watch.

[13] Garmin Customer Support. What is Garmin’s wheelchair mode?.

[14] Moreno D, Glasheen E, Domingo A (2020). Validity of caloric expenditure measured from a wheelchair user smartwatch. Int J Sports Med.

[15] Benning NH, Knaup P, Rupp R (2021). Measurement performance of activity measurements with newer generation of Apple Watch in wheelchair users with spinal cord injury. Methods Inf Med.

[16] Froehlich-Grobe K, Lee J, Ochoa C (2022). Effectiveness and feasibility of the workout on wheels internet intervention (WOWii) for individuals with spinal cord injury: a randomized controlled trial. Spinal Cord.

[17] Rimmer JH, Mehta T, Wilroy J (2019). Rationale and design of a Scale-Up Project Evaluating Responsiveness to Home Exercise And Lifestyle Tele-HEALTH (SUPER-HEALTH) in people with physical/mobility disabilities: a type 1 hybrid design effectiveness trial. BMJ Open.

[18] Tanhoffer RA, Tanhoffer AIP, Raymond J, Hills AP, Davis GM (2012). Comparison of methods to assess energy expenditure and physical activity in people with spinal cord injury. J Spinal Cord Med.

[19] Grigorean VT, Sandu AM, Popescu M (2009). Cardiac dysfunctions following spinal cord injury. J Med Life.

[20] Hiremath SV, Amiri AM, Thapa-Chhetry B (2019). Mobile health-based physical activity intervention for individuals with spinal cord injury in the community: a pilot study. PLoS One.

[21] Nahum-Shani I, Smith SN, Spring BJ (2018). Just-in-Time Adaptive Interventions (JITAIs) in mobile health: key components and design principles for ongoing health behavior support. Ann Behav Med.

[22] SimpleLink™ Bluetooth low energy/multi-standard sensortag design&development info. CC2650STK Development kit | TI.com.

[23] Fogg BJ A behavior model for persuasive design.

[24] Schoeppe S, Alley S, Van Lippevelde W (2016). Efficacy of interventions that use apps to improve diet, physical activity and sedentary behaviour: a systematic review. Int J Behav Nutr Phys Act.

[25] Tsang K (2018). Using Wearable Sensors for Physical Activity Measurement and Promotion in Manual Wheelchair Users. Dissertation.

[26] Brug J, Oenema A, Ferreira I (2005). Theory, evidence and Intervention Mapping to improve behavior nutrition and physical activity interventions. Int J Behav Nutr Phys Act.

[27] Michie S, Abraham C, Whittington C, McAteer J, Gupta S (2009). Effective techniques in healthy eating and physical activity interventions: a meta-regression. Health Psychol.

[28] Shwetar YJ, Veerubhotla AL, Huang Z, Ding D (2020). Comparative validity of energy expenditure prediction algorithms using wearable devices for people with spinal cord injury. Spinal Cord.

[29] Shwetar Y, Huang Z, Veerubhotla A (2022). Predicting physical activity intensity using raw accelerometer signals in manual wheelchair users with spinal cord injury. Spinal Cord.

[30] Veerubhotla A (2019). How Well Can Wearable Devices Measure Physical Activity in Manual Wheelchair Users With Spinal Cord Injury. PhD Thesis.

[31] Pellegrini CA, Hoffman SA, Daly ER, Murillo M, Iakovlev G, Spring B (2015). Acceptability of smartphone technology to interrupt sedentary time in adults with diabetes. Transl Behav Med.

[32] Faul F, Erdfelder E, Buchner A, Lang AG (2009). Statistical power analyses using G*Power 3.1: tests for correlation and regression analyses. Behav Res Methods.

[33] Kroll T, Kehn M, Ho PS, Groah S (2007). The SCI Exercise Self-Efficacy Scale (ESES): development and psychometric properties. Int J Behav Nutr Phys Act.

[34] Cowan RE, Callahan MK, Nash MS (2012). The 6-min push test is reliable and predicts low fitness in spinal cord injury. Med Sci Sports Exerc.

[35] Bangor A, Kortum P, Miller J (2009). Determining what individual SUS scores mean: adding an adjective rating scale. J Usability Studies.

[36] Sauro J MeasuringU. Measuring Usability with the System Usability Scale (SUS).

[37] Lewis JR (2018). The System Usability Scale: past, present, and future. Int J Hum Comput Interact.

[38] Lewis JR, Sauro J (2009). Human Centered Design.

[39] Muhindo M, Bress J, Kalanda R (2021). Implementation of a newborn clinical decision support software (NoviGuide) in a rural district hospital in Eastern Uganda: feasibility and acceptability study. JMIR Mhealth Uhealth.

[40] Kim H, Lee SH, Cho NB, You H, Choi T, Kim J (2020). User-dependent usability and feasibility of a swallowing training mHealth app for older adults: mixed methods pilot study. JMIR Mhealth Uhealth.

[41] Scott AR, Alore EA, Naik AD, Berger DH, Suliburk JW (2017). Mixed-methods analysis of factors impacting use of a postoperative mHealth app. JMIR Mhealth Uhealth.

[42] Skender S, Ose J, Chang-Claude J (2016). Accelerometry and physical activity questionnaires—a systematic review. BMC Public Health.

[43] Kim Y, Ko SH, Lee JL, Huh S (2024). Current status and barriers of exercise in individuals with spinal cord injuries in Korea: a survey. Healthcare (Basel).

[44] Castan A, Úbeda-Colomer J, Chamarro A, Vidal J, Benito-Penalva J, Sauri J (2024). Socio-ecological barriers to leisure time physical activity in Spanish wheelchair users with spinal cord injury: associations with sociodemographic characteristics and functional independence. Arch Phys Med Rehabil.

[45] LaVela SL, Wu J, Nevedal AL (2023). The impact of the COVID-19 pandemic on individuals living with spinal cord injury: a qualitative study. Rehabil Psychol.

[46] Perrier MJ, Latimer-Cheung AE, Ginis KAM, the SHAPE-SCI Research Team (2012). An investigation of seasonal variation in leisure-time physical activity in persons with spinal cord injury. Spinal Cord.

[47] Mason B, Warner M, Briley S, Goosey-Tolfrey V, Vegter R (2020). Managing shoulder pain in manual wheelchair users: a scoping review of conservative treatment interventions. Clin Rehabil.

[48] Adams NT, Tong B, Buren R, Ponzano M, Jun J, Martin Ginis KA (2024). A scoping review of acute sedentary behaviour studies of people with spinal cord injury. Int J Environ Res Public Health.

[49] Karkauskiene E, Tully MA, Dudoniene V (2023). Effectiveness of interventions for reducing sedentary behaviour in older adults living in long-term care facilities: a protocol for a systematic review. Healthcare (Basel).

[50] Harvey LA, Herbert RD (2002). Muscle stretching for treatment and prevention of contracture in people with spinal cord injury. Spinal Cord.

[51] Durmuş A (2024). The influence of digital literacy on mHealth app usability: the mediating role of patient expertise. Digit Health.

[52] McDonald K, Seltzer E, Lu M (2023). Quantifying the impact of the COVID-19 pandemic on clinical trial screening rates over time in 37 countries. Trials.

